# Polyamines Inhibit Porin-Mediated Fluoroquinolone Uptake in Mycobacteria

**DOI:** 10.1371/journal.pone.0065806

**Published:** 2013-06-03

**Authors:** Jansy Passiflora Sarathy, Edmund Lee, Véronique Dartois

**Affiliations:** 1 Novartis Institute for Tropical Diseases Pte Ltd, Singapore, Singapore; 2 Department of Pharmacology, Yong Loo Lin School of Medicine, National University of Singapore Clinical Research Centre, Singapore, Singapore; 3 Department of Biological Sciences, National University of Singapore, Singapore, Singapore; Oregon State University, United States of America

## Abstract

Polyamines decrease the permeability of the outer membrane of *Escherichia coli* to fluoroquinolones and β-lactams. In this study, we tested the effect of four polyamines (spermidine, spermine, cadaverine and putrescine) on fluoroquinolone uptake in *Mycobacterium bovis* BCG. Our results show that polyamines are also capable of reducing the permeability of the mycobacterial outer membrane to fluoroquinolones. Spermidine was most effective and demonstrated reversible dose- and pH-dependent inhibition of ciprofloxacin accumulation. The extent of this inhibition was demonstrated across the fluoroquinolone compound class to varying degrees. Furthermore, we have shown that the addition of spermidine increases the survival of *M. bovis* BCG after a 5-day exposure to ciprofloxacin by up to 25 times. The treatment of actively-replicating *Mycobacterium tuberculosis* with spermidine reduced ciprofloxacin accumulation by half while non-replicating nutrient-starved *M. tuberculosis* cultures lacked similar sensitivity to polyamines. Gene expression studies showed that several outer membrane proteins are significantly down–regulated during the shift to non–replication. Collectively, these characteristics of fluoroquinolone uptake in *M. bovis* BCG are consistent with facilitated transport by porin-like proteins and suggest that a reduction in intracellular uptake contributes to the phenotypic drug resistance demonstrated by *M. tuberculosis* in the non-replicating state.

## Introduction

Tuberculosis is an international health concern with little foreseeable relief [1]. With the emergence of multi-drug resistance (MDR) of the causative agent, *Mycobacterium tuberculosis*, comes the need to understand the molecular basis of resistance acquisition. Antibiotic penetration of the notoriously impermeable mycobacterial outer membrane requires further exploration. Several mycobacterial efflux pumps have already been associated with reduced susceptibility to anti-tuberculous agents such as isoniazid, tetracycline, fluoroquinolones and aminoglycosides [2]. Antibiotic uptake processes, however, are less clearly understood. Fluoroquinolones form a recent class of drugs increasingly used in the fight against tuberculosis, with members such as moxifloxacin, ofloxacin and levofloxacin being used as second-line treatment agents. While Rv2686-Rv2687-Rv2688c and LfrA have been associated with fluoroquinolone efflux activity in mycobacteria [3], [4], it remains unclear how fluoroquinolone uptake occurs. The observation that the activity of fluoroquinolones against mycobacteria increases with the hydrophobicity of the drug indicates the preference for direct diffusion across lipid membranes [5]. However, a study of *M. smegmatis* mutants carrying deletions of *mspA* and *mspC,* which code for the porins MspA and MspC respectively, showed a 4-fold decrease in intracellular accumulation of norfloxacin compared to the wild-type [6]. It was concluded that porins play an important role in the uptake of hydrophilic fluoroquinolones in mycobacteria.

Polyamines are naturally-occurring organic molecules with at least two primary amino-groups. Endogenous to both prokaryotic and eukaryotic cell types, these polycations are produced via complex pathways involving the decarboxylation of ornithine, arginine and lysine. Although their exact functions are unclear, they are understood to play pleiotropic roles in cell growth and survival [7]. The effects of four polyamines (spermine, spermidine, cadaverine and putrescine) on the activity of *E. coli* porins OmpC and OmpF have been documented. These polyamines were shown to suppress channel opening, enhance channel closure as well as promote the inactive state [8]. It has been suggested that the main mechanism of porin channel modulation involves changes in the intrinsic rate constants for gating, which leads to the stabilization of the closed states. A handful of studies have looked at the *in vitro* effects of polyamines on the accumulation of antibiotics in *E. coli*. Spermidine has been shown to reduce norfloxacin and β-lactam diffusion through OmpF, while cadaverine has been shown to reduce ampicillin susceptibility in *E. coli* [9]–[11]. We hypothesize that polyamines may reduce the overall penetration rate of these agents in mycobacteria as well.

Several studies have demonstrated that non-replicating nutrient-starved *M. tuberculosis* displays phenotypic resistance to multiple standard anti-tuberculous agents [12]–[14]. Such resistance is the result of metabolic and physiological adaptation of the bacilli to nutrient-depletion, and is not genetically determined. Full drug susceptibility is re-established upon the resumption of bacterial replication. Fluoroquinolones such as moxifloxacin, ofloxacin and gatifloxacin have minimum bactericidal concentrations that range between 0.25–2.5 µM against replicating *M. tuberculosis*, but greater than 100 µM against nutrient-starved cultures [13], [14]. The reduction in drug susceptibility that occurs upon the onset of dormancy is thought to be the result of reorganization of the outer membrane [15]. To understand fluoroquinolone transport across the mycobacterial outer membrane, we have investigated the uptake and activity of this drug class in *M. bovis* BCG, using microbiologic and liquid-chromatography mass-spectrometric (LCMS) methods.

In the present work, we first test the hypothesis that, as seen in *E. coli,* polyamines inhibit the intracellular accumulation of the fluoroquinolones in BCG, resulting in decreased anti -mycobacterial activity. We then demonstrate that this effect is reduced in non-replicating *M. tuberculosis*, suggesting that reduced fluoroquinolone permeability of the outer membrane may contribute to the development of phenotypic drug resistance. Through the use of various uptake assay conditions, we show that efflux pumps do not affect intracellular concentrations of the fluoroquinolones, and propose that porins might constitute a valid route of cell entry for this drug class.

## Materials and Methods

### Chemicals, Strains and Growth Conditions

Spermidine, spermine, cadaverine and putrescine were obtained from Sigma-Aldrich (Missouri, U S A). Stock solutions of 1M were prepared for all polyamines. Ofloxacin, verapamil and reserpine were obtained from Sigma Aldrich, moxifloxacin, gatifloxacin and linezolid from Sequoia Research Products (UK), and ciprofloxacin and rifampicin from Fluka (Missouri, U S A). Stock solutions of 10 mM were prepared for these compounds and stored at 4°C. Phosphate-buffered saline (PBS) was provided by Invitrogen (Carlsbad, California). HPLC-grade methanol and acetonitrile were purchased from Fisher Scientific (Hampton, New Hampshire).

*M. bovis* BCG and *M. tuberculosis* H37Rv were cultured in Middlebrook 7H9 broth (provided by Becton, Dickinson and Company, New Jersey, U S A) supplemented with 0.4% ADS, 0.2% glycerol and 0.05% Tween80 or grown on Middlebrook 7H11 agar (Becton, Dickinson and Company) supplemented with 10% OADC and 0.5% glycerol. Broth cultures were incubated at 37°C till an OD_600_ of 0.3–0.4 was reached. The addition of sodium hydroxide was necessary in experiments where the pH of supplemented 7H9 media was increased. Nutrient-starved non-replicating *M. tuberculosis* cultures were generated by re-suspending exponentially-growing H37Rv in PBS and incubating at 37°C with constant rolling for 14 days [12], [13]. Agar plates were incubated at 37°C and bacterial colonies were counted after 2–3 weeks.

### Antibiotic penetration assay

A drug penetration assay was optimized for the measurement of steady-state intracellular drug accumulation in *M. bovis* BCG and *M. tuberculosis*. Broth cultures were harvested by centrifugation at 3,200 g for 10 min. The pellets were re-suspended in fresh 7H9 broth media or PBS-Tween80 to an OD_600_ of 4.0. Serial dilutions of this concentrated culture were grown on 7H11 agar plates to enable determination of cell density for each drug penetration assay. Unless otherwise mentioned, all standard drug penetration assays were conducted at a drug incubation concentration of 10 µM, for an incubation period of 30 min. This corresponds with 3.3 µg/ml ciprofloxacin, 4.0 µg/ml moxifloxacin, 3.6 µg/ml ofloxacin, 3.8 µg/ml gatifloxacin, 8.2 µg/ml rifampicin, 3.4 µg/ml linezolid and 2.0 µg/ml ethambutol. Three 300 µl aliquots were pelleted at 13,200 g for 3 min at 4°C. The pellets were washed in an equal volume of PBS. The cells were then lysed overnight in an equal volume of glycine-HCl (pH 3) at 37°C with constant agitation [6], [16]._ENREF_4 Several published lysis methods were tested by comparing drug recovery, matrix effects and quantitation limits by HPLC/MS/MS. This procedure was most effective at achieving complete lysis of mycobacterial cells for the purpose of LCMS quantitative analysis. Cell lysates were filtered with 0.22 µm filter units (Millipore MILLEX-GV filter units, PVDF, 13 mm) to ensure sample sterility. Compound extraction was achieved by the addition of 80 µl of methanol and 40 µl of acetonitrile. Lysate samples were subsequently stored at −20°C. In instances where the time-course of drug accumulation was examined over a 10 min incubation period, duplicate 300 µl aliquots were removed at regular time intervals.

In efflux pump inhibition experiments, 75 µM verapamil and 20 µM reserpine were added (34.1 µg/ml and 12.2 µg/ml respectively) 3 min after the addition of the anti-tuberculous agent (10 µM). In uptake inhibition experiments, cultures were pre-incubated with the specified polyamine for a period of 10 min at 37°C at a concentration of 10 mM unless otherwise mentioned. This corresponds with 1.5 mg/ml spermidine, 2.0 mg/ml spermine, 1.0 mg/ml cadaverine and 0.9 mg/ml putrescine.

### LCMS analytical methods

Quantification of drug concentration was achieved by liquid chromatography - mass spectrometry (LC/MS). Mass analysis and detection were performed on an API 4000 Q-trap triple-quadrupole mass spectrometer (Applied Biosystems,) equipped with a turbo ion-spray ionization source. The HPLC system is of the Agilent (USA) 1200 series with a degasser, binary pump, autosampler and thermostated column compartment. Chromatographic separation was achieved with a Gemini C18 column for the three fluoroquinolones and a Gemini C6-Phenyl column for all other drugs (150×4.6 mm, 5 µm; Phenomenex). Mobile phases, water and acetonitrile, were acidified with 0.1% acetic acid. All fluoroquinolones were eluted with 3% to 40% linear gradients of acidified acetonitrile at a flow rate of 0.8 ml/min; rifampicin, ethambutol and linezolid were eluted with 3% to 50% linear gradients. Calibration standards for all drugs were prepared from stock solutions to give final concentrations of 5 nM to 500 nM. Limits of quantitation (LOQs) for all drugs range from 0.5 to 10 nM.

### Determination of cellular drug accumulation and statistical testing

In instances when drug accumulation was compared between two individually conducted penetration assays, cell lysate drug concentrations were multiplied by the solvent-crashed lysate volume and normalized by the number of CFU per 300 µl aliquot. The result is an expression of the amount of drug (nmol) per bacterial unit. When drug accumulation was being compared within single penetration assays, cell lysate concentrations (nmol/dm^3^) were used as is without normalization. All statistical comparisons were performed with unpaired two-sample *t*-tests. Statistical significance was set at *p* values of <0.01 and <0.001 (highly significant).

### Susceptibility tests

*M. bovis* BCG broth cultures were diluted to an OD_600_ of 0.02 and 200 µl aliquots were transferred to 96-well plates. For the determination of polyamine MICs (concentrations at which 90% of bacterial growth was inhibited), dilutions of spermidine and cadaverine were spotted to achieve final concentrations between 0.001 and 50 mM. In MIC-shift experiments, dilutions of ciprofloxacin were prepared to achieve the final range of 0.05-2.0 µM; spermidine and cadaverine were co-spotted with ciprofloxacin when needed to achieve the final concentrations of 0.01, 0.1 and 1 mM. All 96-well plates were incubated at 37°C, and their optical densities were read after 5 days. In order to investigate the killing of *M. bovis* BCG in broth by 10 mM spermidine over a 60 min period, aliquots removed every 20 min were grown on drug-free agar plates following ten-fold serial dilutions and compared to a control (drug-free) culture. In a separate kill kinetics assay demonstrating changes in ciprofloxacin bactericidal activity, broth cultures were incubated with 1 µM ciprofloxacin alone and in combination with 1 mM or 2.5 mM spermidine over a five-day period; aliquots removed on days 1, 2 and 5 were similarly grown on drug-free agar plates.

### Total RNA extraction

Total RNA was isolated from replicating and non–replicating *M. tuberculosis* cultures using the Trizol method. Briefly, cultures were grown to an OD_600_ of 0.4–0.5 and harvested by centrifugation. Pellets were re-suspended in Trizol and bead-beaten at 6,500 rpm to achieve homogenization. Lysates were centrifuged and 300 µl of chloroform: isoamyl alcohol (24∶1) was added to the supernatant. After 15 min incubation at room temperature, samples were centrifuged at 12,000 rpm for 15 min. 0.9 volumes of isopropanol was added to the aqueous phase and incubated at room temperature for 10 min. RNA precipitate was pelleted at 4°C, washed with 75% ethanol and stored at −80°C. RNA purification was performed with the RNeasy Mini Kit (Qiagen; California, U.S.A.) as directed by the user manual. DNase digestion of the total RNA extract was performed using the the RNase-Free DNase Set (Qiagen; California, U.S.A.). Approximately 1 µg of RNA was incubated with 0.5Kunitz units of DNase for 30 min at 37°C as directed. DNase was inactivated with EDTA and incubation for 5 min at 65°C.

### Quantitative RT-PCR

A pair of primers was designed for each of the ten *M. tuberculosis* outer membrane proteins (OMPs) analysed in this study and listed in Table 1. *16s rRNA* was included as a positive control. cDNA synthesis from total RNA extracts was performed using the iScript Select cDNA Synthesis Kit and random primers (BioRad; California, U.S.A.) as directed by the user manual. Quantitative RT-PCR was performed by the iQ5 Real Time Detection System (BioRad; California, U.S.A.) using the SYBR Green Supermix Kit (BioRad). The amplification method consisted of 40 cycles of 95°C for 30 s, 61°C for 30 s and 72°C for 30 s. Analysis was done in triplicates and the experiment was conducted twice. No amplification controls (NACs) excluding reverse transcriptase were prepared for each gene to check for DNA contamination. RT-PCR products were analysed by agarose (1.0% wt/vol) gel electrophoresis. No products were obtained with NAC reactions. Relative gene expression data was expressed using the 2^−ΔΔCT^ method as detailed by Livak and Schmittgen [17].

**Table 1:** Primer pairs used for qRT-PCR analysis of OMP gene expression in *M. tuberculosis*.

Gene name	Direction	Oligonucleotide sequence (5^1^ – 3^1^)
*rv0899 (ompATb)*	Forward	A C C G T T A C T C T G A T C G G T G A C T
	Reverse	G A G A A A T C A A G T G A T C G C A C A A
*rv1698*	Forward	G G T C T C A T T G A C C C A G G A G T T
	Reverse	A G T T T G G T G C T C A A C T G G C T A C
*rv1973*	Forward	G A C G C C T A C A C A C A G C T G A C
	Reverse	T G A T G G T C T G G T T T A C G A A C A G
*rv0227*	Forward	C C G A G A A G A A G A C A T A C C C C T A
	Reverse	C G G T A T G T G G T T A A A C C G T T G
*rv0431*	Forward	T C T A C A A C A T C T C A G G C A C A G A
	Reverse	G A C G T C G G G T A A C G A T A G A T T C
*rv1351*	Forward	C T G G G T G T T A T T G G C T T G C T C G
	Reverse	T C A A G T A C C T A T G A C G G T G C T G
*rv1352*	Forward	C G A A T C C C C T T A T T T T G G T G T
	Reverse	T T G G T G T C G A C A A T G T T C T C A T
*rv1968*	Forward	C T A T C C G G T G G G A A A A G T G T C
	Reverse	A C A A G C C C T T G G T T T T G A T T G
*rv1970*	Forward	C C C G A A C G A G A C G T T C C A A A A T
	Reverse	G A G C T G G G T C A G A T G A C A C T C
*rv2270*	Forward	A C G A A C A T G A A T C C G A C A A A C
	Reverse	T T G A A G G T T A A T C C A G G T C T C G
*16s rRNA*	Forward	G G A C A C C T A T T A C G A T C A C C A G
	Reverse	C A A A A C C T C A T C G G A A T C A C G

## Results

### Intracellular drug accumulation

**Figure 1:**
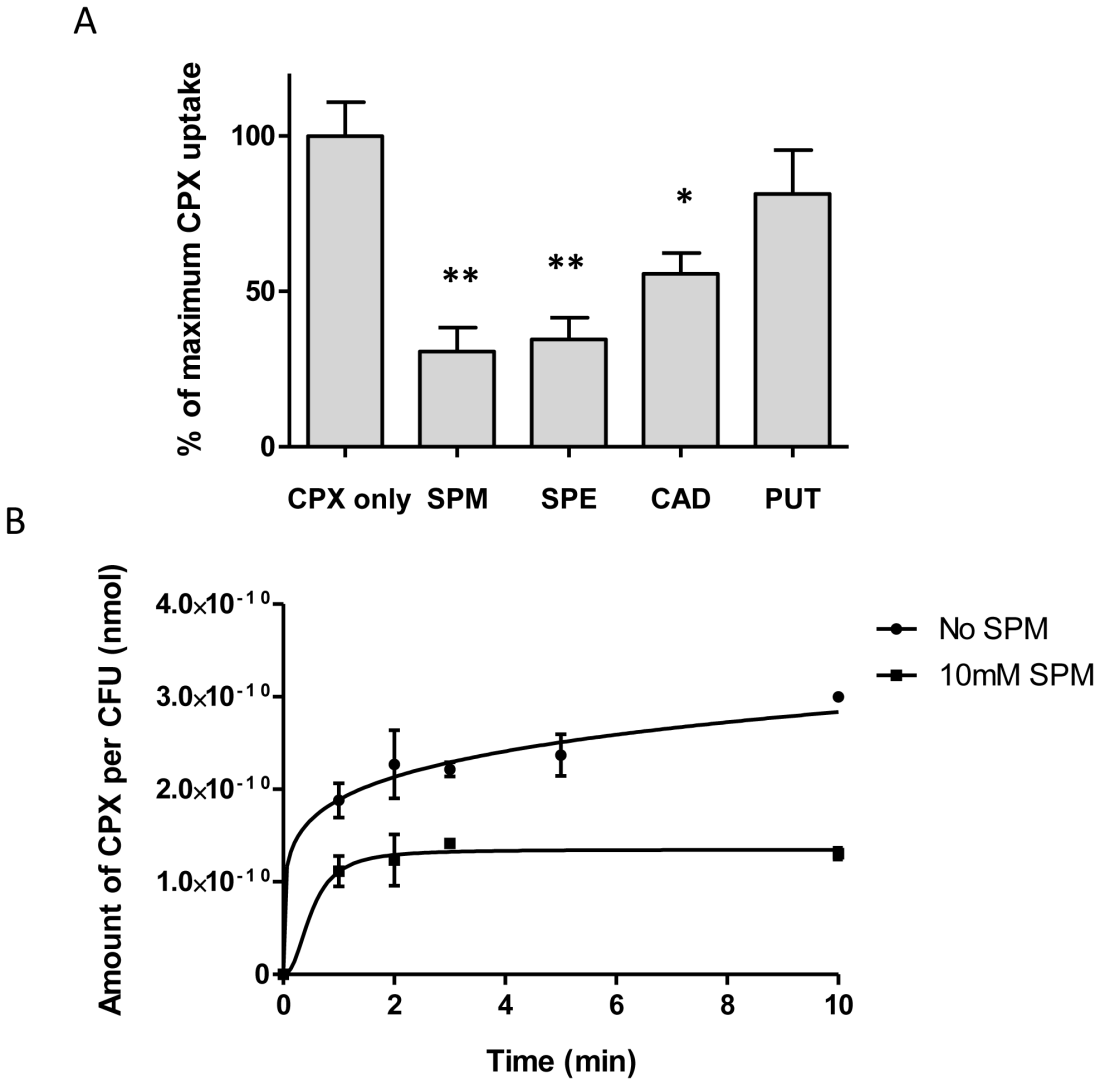
Effects of polyamines on ciprofloxacin accumulation in *M. bovis* BCG. (A) Inhibition of ciprofloxacin (CPX) accumulation following treatment with 4 different polyamines. Relative accumulation of CPX is expressed as a percentage of average uninhibited accumulation. Experiments were done in biological triplicates. Asterisks denote data points that differed significantly between polyamine-treated and -untreated BCG. (**, p <0.001; *, p <0.01). (B) Time-course of CPX accumulation by spermidine-treated and -untreated BCG. Data was normalized against bacterial counts for each experiment. Results are expressed as the amount of CPX (nmol) per CFU. Experiments were done in biological duplicates. Standard deviations are shown as error bars.

To investigate the effect of polyamines on fluoroquinolone accumulation in *M. bovis* BCG, intracellular concentrations of ciprofloxacin were measured in exponentially growing cultures in the presence of spermidine, spermine, cadaverine or putrescine. The polyamine incubation concentration of 10 mM was chosen in light of published work on porin inhibition by polyamines in *E. coli* [10], [18]. Spermidine, spermine, and cadaverine significantly reduced intracellular accumulation of ciprofloxacin in *M. bovis* BCG (Figure 1A). At 10 mM (1.5 mg/ml), spermidine proved most potent at decreasing ciprofloxacin accumulation, reducing the final ciprofloxacin intracellular accumulation by 69% relative to the control assay (*p* <0.001). Hence, spermidine was chosen for subsequent experiments. Putrescine was least effective, causing an apparent reduction in steady-state accumulation which lacked statistical significance. The accumulation of ciprofloxacin was examined over the course of the first 10 min in the presence of 10 mM spermidine. Most of the inhibitory effect was observed within the first minute. While non-treated *M. bovis* BCG continued to accumulate ciprofloxacin after the first 10 min of incubation, the spermidine-treated cells reached steady-state accumulation within 3 min (Figure 1B).

**Figure 2:**
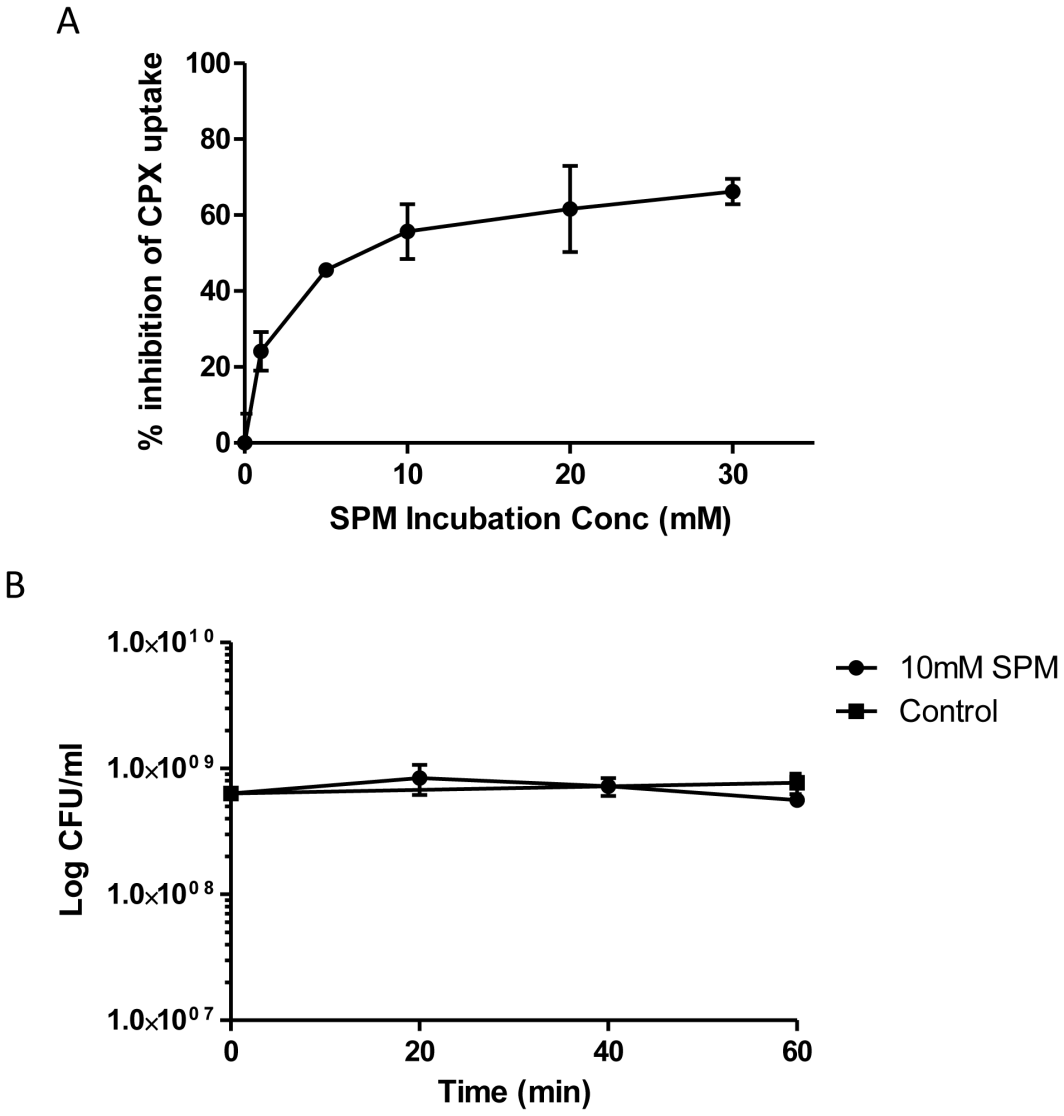
Dose-dependency of spermidine-induced inhibition of intracellular accumulation. (A) Plot of percentage inhibition of ciprofloxacin (CPX) accumulation by *M. bovis* BCG in response to increasing concentrations of spermidine (SPM). This experiment was done in biological triplicates. (B) Kill -kinetics of 10 mM of SPM against *M. bovis* BCG for the first 60 min of incubation. This experiment was done in biological duplicates. Standard deviations are shown as error bars.

Increasing doses of spermidine from 1 mM to 30 mM (0.15 mg/ml to 4.5 mg/ml) produced a continuous decrease in steady-state accumulation of ciprofloxacin. Figure 2A shows that this spermidine-induced inhibition is dose dependent, with an IC_50_ of about 7 mM (1.05 mg/ml). It appears that maximum inhibition is approached at spermidine concentrations greater than 30 mM. In order to ensure that any reduction in drug accumulation by *M. bovis* BCG brought about by spermidine at 10 mM was not the result of cell death, a kill kinetics experiment was conducted. After a 60 min incubation period, 10 mM of spermidine did not significantly reduce the number of colony-forming units (Figure 2B).

**Figure 3:**
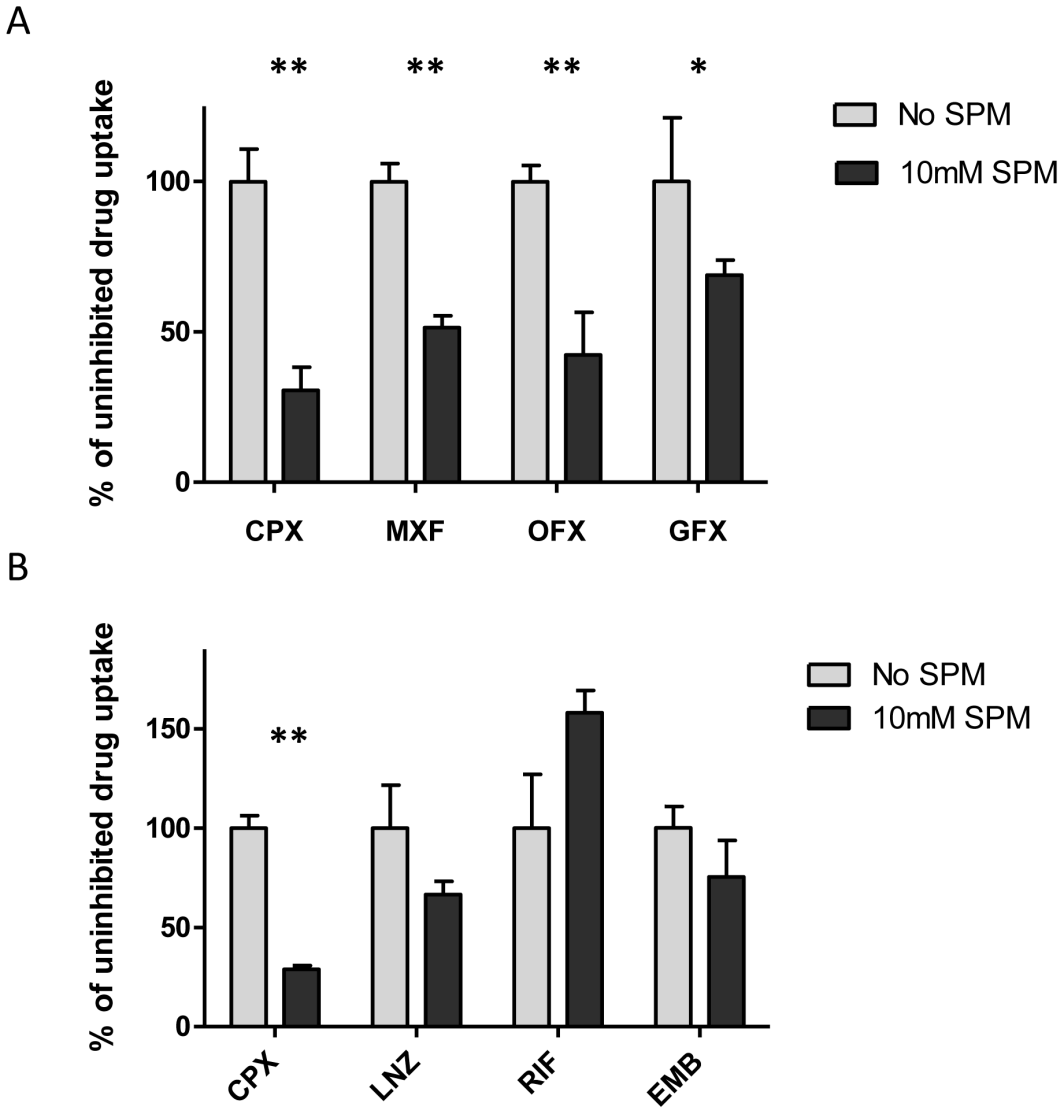
Effects of spermidine on the accumulation of fluoroquinolone and non-fluoroquinolones in *M. bovis* BCG. (A) Inhibition of moxifloxacin (MXF), ofloxacin (OFX) and gatifloxacin (GFX), and ciprofloxacin (CPX) accumulation in BCG by spermidine (SPM). (B) Inhibition of linezolid (LNZ), rifampicin (RIF) and ethambutol (EMB) accumulation in BCG by SPM. Relative accumulation of each drug is expressed as a percentage of uninhibited drug accumulation respectively. Experiments were done in biological triplicates. Standard deviations are shown as error bars. Asterisks denote data points that differed significantly between SPM-treated and -untreated BCG. (**, p <0.001; *, p <0.01).

Reductions in moxifloxacin, ofloxacin and gatifloxacin steady-state intracellular accumulation were also observed in the presence of spermidine, proving that this phenomenon is consistent across the fluoroquinolone class (Figure 3A). The extent of reduction in accumulation varied between 69% and 31% across the four fluoroquinolones, with ciprofloxacin accumulation displaying the greatest reduction. In order to investigate if the effect of polyamine pre-treatment on drug uptake is fluoroquinolone-specific, further experiments were conducted with ethambutol, rifampicin and linezolid. The presence of 10 mM spermidine resulted in a 33% and 25% reduction in steady-state accumulation of linezolid and ethambutol respectively (Figure 3B). In contrast, rifampicin displayed an increase in accumulation by 58% in the presence of spermidine. The apparent increase in intracellular accumulation of rifampicin further supports the notion that the reduction of accumulation seen with other agents is not an artifact due to decreased cell viability caused by the 10 mM spermidine treatment.

### Effect of spermidine on mycobacterial susceptibility to ciprofloxacin

**Figure 4:**
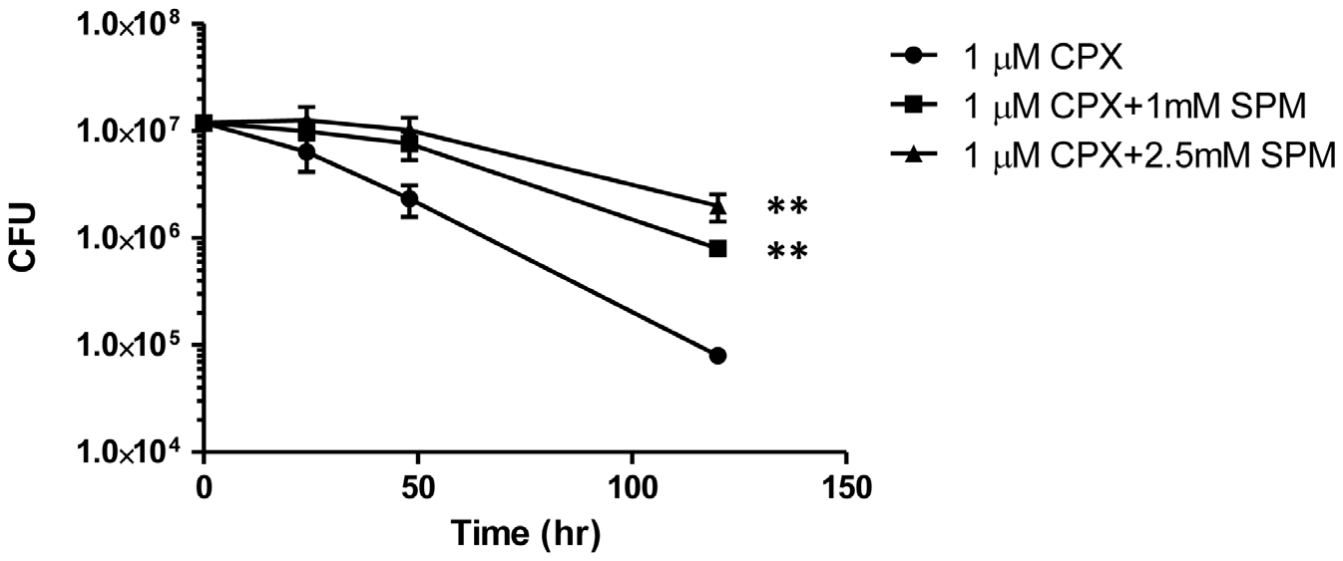
Survival of *M. bovis* BCG treated with ciprofloxacin in the presence of spermidine. The effects of pre-incubation with 2.5 mM and 1 mM of spermidine (SPM) were compared against incubation with 1 µM ciprofloxacin (CPX) alone. The CFU counts are plotted on a log scale. Experiments were done in biological duplicates. Standard deviations are shown as error bars. Asterisks denote data points that differed significantly between SPM-treated and -untreated BCG. (**, p <0.001).

Given the reduced uptake of ciprofloxacin in the presence of polyamines, we hypothesized that the bactericidal activity of ciprofloxacin could be weakened in the presence of spermidine. We first tested the growth inhibitory properties of spermidine and cadaverine individually. The two polyamines completely inhibited growth at about 4 mM and 8 mM, respectively. At 0.01, 0.1 and 1.0 mM, neither polyamine brought about a shift in ciprofloxacin-mediated growth inhibition; the MIC of ciprofloxacin against *M. bovis* BCG remained consistent at 0.7 µM. However, enumeration of colony-forming units revealed that ciprofloxacin-mediated killing of *M. bovis* BCG was reduced by 10 and 25-fold in the presence of 1.0 mM and 2.5 mM spermidine respectively over a 5 day period (Figure 4). At 1 µM, ciprofloxacin caused a 2-log reduction in the number of viable bacilli, while less than 1-log reduction was observed in the presence of 2.5 mM spermidine. The differences in the number of viable bacilli between spermidine-treated and untreated cultures are statistically significant at the 5-day mark (*p*<0.001).

### Effect of spermidine on ciprofloxacin uptake in non-replicating *M. tuberculosis*

**Figure 5:**
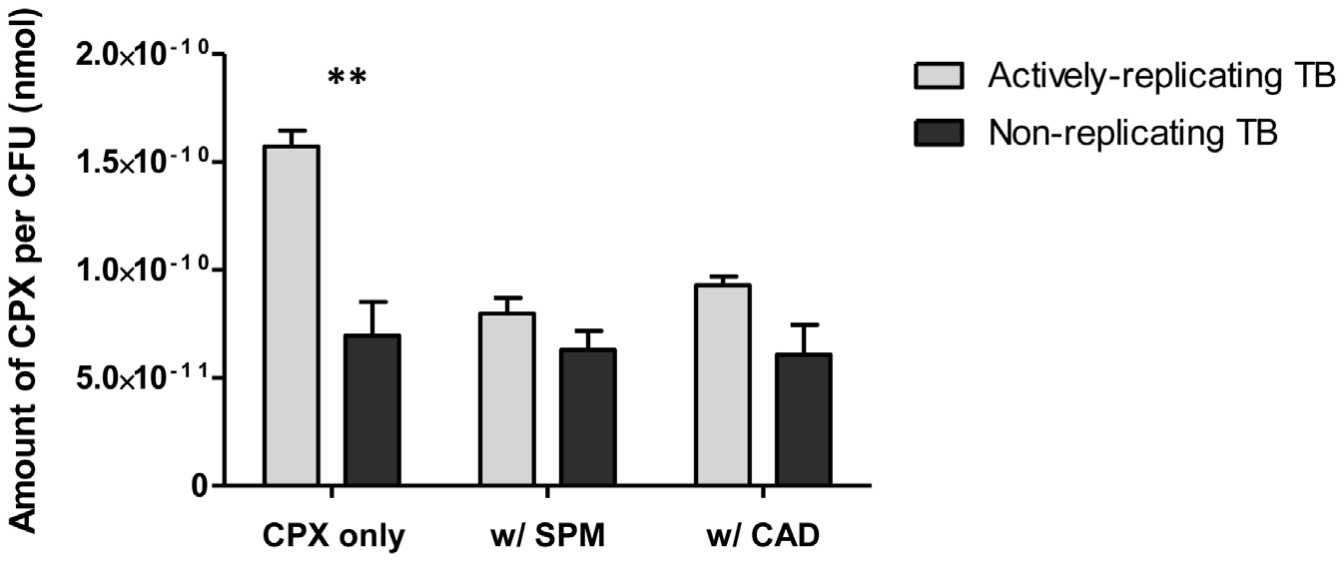
Effects of polyamines on ciprofloxacin accumulation in non-replicating *M. tuberculosis*. Data was normalized against bacterial counts for each experiment. Results are expressed as the amount of ciprofloxacin (CPX) (nmol) per CFU. Experiments were done in biological triplicates. Standard deviations are shown as error bars. Asterisks denote significant differences between replicating and non-replicating *M. tuberculosis* (**, p <0.001).

Since fluoroquinolones have significantly reduced activity against non-replicating *M. tuberculosis*, under conditions of nutrient starvation or hypoxia [13], we next asked whether reduced intracellular uptake could contribute to this observation. Non-replicating cultures of *M. tuberculosis* were generated by starving the cells of nutrients over a two-week incubation period. Ciprofloxacin penetration assays were performed on replicating and non-replicating cultures in a similar manner and results were expressed as the amount of ciprofloxacin per colony-forming unit. A comparison of uninhibited ciprofloxacin accumulation between the two cultures, as shown in Figure 5, shows that absolute ciprofloxacin content of replicating *M. tuberculosis* was 2.3-fold greater (*p*  = 0.001) than that of non-replicating cultures. To determine whether the polyamine-inhibited mechanism of fluoroquinolone uptake played a role in non-replicating cells, we compared the accumulation of ciprofloxacin in replicating and non-replicating cultures, with and without spermidine or cadaverine. At 10 mM, the polyamines produced 49% and 41% reduction in ciprofloxacin accumulation in replicating *M. tuberculosis,* respectively. In the non-replicating culture however, spermidine and cadaverine decreased accumulation by only 9% and 13% respectively, indicating a reduced contribution of the polyamine-inhibited transport pathway to ciprofloxacin uptake in the starvation-induced non-replicating state.

### Characteristics of fluoroquinolone uptake in *M. bovis* BCG

#### Reversibility of effects of polyamines

**Figure 6:**
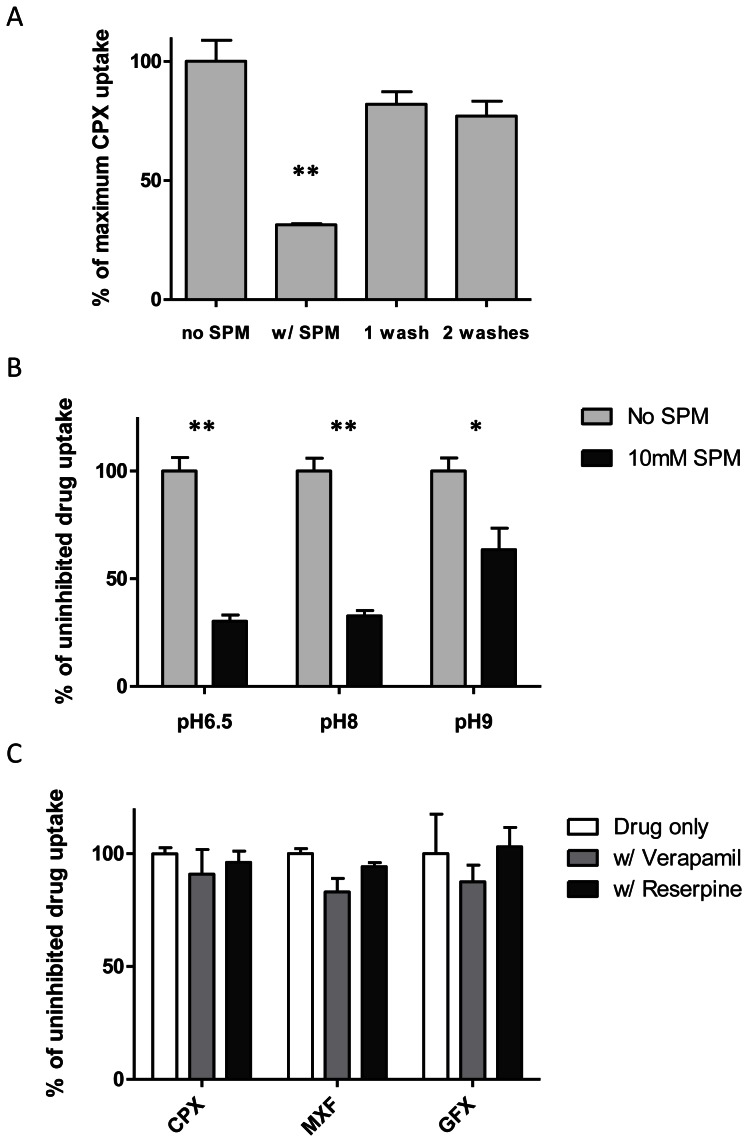
Characteristics of fluoroquinolone accumulation in *M. bovis* BCG. (A) Effect of PBS washes on ciprofloxacin (CPX) accumulation in BCG that has been pre-incubating with spermidine (SPM). Relative CPX accumulation was expressed as percentages of uninhibited accumulation. (B) Effect of increasing pH on the inhibitory effects of SPM. CPX accumulation in the presence of SPM was normalized against uninhibited accumulation for the each pH condition respectively. (C) Effect of efflux pump inhibitors, verapamil (75 µM) and reserpine (20 µM), on CPX, moxifloxacin (MXF) and gatifloxacin (GFX) accumulation in BCG. Relative accumulation of each drug is expressed as the percentage of uninhibited accumulation respectively. Experiments were done in biological triplicates. Standard deviations are shown as error bars. Asterisks denote significant differences between inhibited and uninhibited reactions (**, p <0.001; *, p <0.01).

The reversibility of the effects of polyamine treatment was investigated to gain insight into the observed inhibitory mechanism. When *M. bovis* BCG cultures were pre-incubating with 10 mM (2.5 times the MIC) spermidine and washed with PBS prior to incubation with ciprofloxacin, a partial recovery in steady-state ciprofloxacin accumulation was observed (Figure 6a). Saline washes returned ciprofloxacin accumulation to approximately 80% of uninhibited accumulation. No significant difference resulted from washing the bacilli twice rather than once. These results indicate that inhibition of fluoroquinolone accumulation by polyamines is mostly reversible. It is possible that a fraction of spermidine remains tightly adhered to the outer membrane despite two PBS washes. Alternatively, recovery in ciprofloxacin accumulation may be underestimated at each additional wash step due to the partial loss of bacilli.

#### Effect of pH changes

It has been reported that the inhibitory effect of cadaverine on cellular permeability in *E. coli* is relieved at higher pH where fewer polyamine molecules are charged [18]. Ciprofloxacin penetration assays were performed on *M. bovis* BCG in pH-adjusted media in order to demonstrate the effect of increasing pH on the inhibitory activity of polyamines. The effects of spermidine on steady-state ciprofloxacin accumulation in BCG were normalized against uninhibited ciprofloxacin accumulation for each pH condition because these pH changes exerted their own effects on the kinetics of ciprofloxacin uptake. Figure 6b shows that at pH 8 spermidine reduced ciprofloxacin accumulation by 68%, which is comparable to results obtained with unadjusted media (pH 6.5). At pH 9, spermidine only produced a 36% reduction in ciprofloxacin accumulation. Cell viability remained unaffected for the incubation duration of 30 min at pH 9 (results not shown).

#### Efflux pump inhibition

To determine whether active efflux influences intracellular accumulation of fluoroquinolones, the penetration of ciprofloxacin, moxifloxacin or gatifloxacin into *M. bovis* BCG was examined in the presence of known efflux pump inhibitors. Verapamil (calcium channel blocker) and reserpine (P-glycoprotein inhibitor) are commonly used efflux pump inhibitors [4], [19], [20]. Previously conducted experiments on *M. bovis* BCG (data not published) indicated that 30 min is sufficient to achieve steady-state intracellular drug accumulation for the range of standard anti-tuberculous agents tested in this study. Neither verapamil nor reserpine, at concentrations of 75 µM and 20 µM respectively, was able to produce a significant change in the steady-state accumulation of ciprofloxacin, moxifloxacin or gatifloxacin in the wild-type strain (Figure 6c).

### qRT-PCR analysis of OMP gene expression

The reversibility and pH dependency of polyamine inhibition demonstrated above, together with prior knowledge of polyamines inhibiting the OmpC and OmpF porins in a dose- and pH-dependent manner in *E. coli* [10], [18], led us to explore the possibility that facilitated transport through porin-like channels could contribute to fluoroquinolone uptake. If this is indeed the case, these transporters would be expected to play a reduced role in non-replicating cells in order to explain our observations. Unfortunately, our knowledge of mycobacterial porins is limited. MspA, the one well-known porin of *M. smegmatis*, is understood to share its amphiphilic beta-barrel structure and potential to be secreted with OMPs of Gram-negative bacteria. Based on these properties, bioinformatics approaches have led to the prediction of several OMPs of *M. tuberculosis* [21], [22]. Quantitative RT-PCR assays were performed to measure the transcript levels of ten OMP genes in actively replicating and nutrient starved non-replicating bacteria. Table 2 shows results obtained from the quantification of expression levels of *ompATB (rv0899), rv1698, rv1973, rv0227, rv0431, rv1351, rv1352, rv1968, rv1970* and *rv2270.* The housekeeping gene *16srRNA* was used as the internal control to normalize the results. Data are presented as expression levels for each gene relative to the *16srRNA* transcript. Transcription levels of actively replicating *M. tuberculosis* were set as the calibrator for the 2^−ΔΔCt^ method. An effect on gene expression levels was considered significant when the corresponding fold-change ratios were ≤−2.5 or ≥2.5. Of the ten OMP genes tested, only *rv1698*, *rv1973* and *rv0431* displayed significant reduction in expression levels in non-replicating bacteria. Specifically, *rv1698* and *rv0431* expression was reduced by ≥25-fold. *OmpATb* and *rv2270* expression levels were increased by 3.0- and 5.1- fold respectively. The expression of *rv1352* was drastically increased by over 1000-fold in non-replicating bacteria. This was the largest change in expression level observed from all the OMP genes tested.

**Table 2:** Differential expression of OMP genes in *M. tuberculosis* during non-replication.

Locus	Gene name	ΔCt (mean±SD)	ΔΔCt (mean±SD)	2^−ΔΔCt^	Fold induction
Rv0899	*ompATb*	−0.47±0.36	−1.59±0.39	3.01	3.0
Rv1698	*mctB*	1.11±0.41	4.74±0.48	0.04	−25.0
Rv0227		−1.51±0.31	2.03±0.41	0.24	−4.2
Rv0431		4.56±0.40	5.21±0.53	0.03	−33.3
Rv1351		0.15±0.30	0.8±0.52	0.57	−1.75
Rv1352		−3.35±0.06	−10.24±0.17	1209.34	1209.3
Rv1968	*mce3C*	2.52±0.18	−1.22±0.27	2.33	2.3
Rv1970	*mce3E*	2.85±0.37	0.68±0.41	0.62	−1.6
Rv1973		2.97±0.23	−1.18±0.31	2.27	2.3
Rv2270	*lppN*	2.28±0.11	−2.36±0.30	5.13	5.1

Relative expression of each gene was calculated according to the 2^−ΔΔCt^ method using *16s rRNA* as the internal control and the expression levels of actively-replicating cultures as the calibrator. Significant fold-changes (≤−2.5 or ≥2.5) were bolded.

## Discussion

In this study, we have explored the possibility of chemically inhibiting fluoroquinolone transport in mycobacteria by use of polyamines. Polyamines have been shown to reduce the outer membrane permeability of *E. coli* to compounds such as fluoroquinolones and β-lactams [9], [18], [8]. Similarly, we have demonstrated that significant reduction of mycobacterial outer membrane permeability is achieved by polyamines in the millimolar range. In *E. coli,* the intracellular concentration of spermidine is around 6 mM. Under normal conditions, the intracellular concentration of putrescine is 20 mM, with the potential to be further increased at high pH [23]. Spermidine-induced inhibition of ciprofloxacin accumulation demonstrated dose dependency up to 30 mM at which point this inhibition leveled off at about 70%. This suggests that fluoroquinolones accumulate intracellularly via more than one pathway, one of which appears to be sensitive to polyamine treatment. The residual uptake, which amounts to around 30% in the case of ciprofloxacin, could be unassisted passive diffusion through the hydrophobic core of the outer membrane. Our data suggest that the extent of dependence on either pathway in mycobacteria differs between fluoroquinolones. As seen with ciprofloxacin, ofloxacin and gatifloxacin, greater hydrophilicity results in more dependence on the polyamine sensitive transport pathway (Table 3). Moxifloxacin, being the most hydrophobic of the group, is likely most effective at diffusing through the lipid-rich membrane, as has also been demonstrated previously in *M. smegmatis* [6].

**Table 3:** Physicochemical properties of fluoroquinolones.

Fluoroquinolone	Mol. Weight	ClogP	PSA	% Decrease
**Ciprofloxacin**	331.35	−0.725	77.04	69%
**Moxifloxacin**	437.9	−0.082	86.27	49%
**Ofloxacin**	361.4	−0.508	77.48	58%
**Gatifloxacin**	375.4	−0.266	86.27	31%

Molecular weights, Partition Coefficients (ClogP), Polar Surface Areas (PSA) and relative spermidine-induced reductions in intracellular accumulation of four fluoroquinolones (as illustrated in Figure 3A).

Our observation of the first 10 min of ciprofloxacin uptake in *M. bovis* BCG showed that treatment with spermidine reduced steady-state intracellular accumulation rather than delaying it. Similar results were obtained in the study of norfloxacin and cefepime uptake in *Enterobacter cloacae* [9]. It appears that following inhibition of drug uptake, the intracellular/extracellular concentration gradient ultimately drives the equilibration of drug concentration between the two compartments. We suggest a cellular model where uptake and efflux processes work in concert to achieve steady-state conditions. A reduction in net steady-state accumulation without impeding efflux processes suggests that polyamines reduce the extent of fluoroquinolone influx. Efflux pump inhibitors verapamil and reserpine were not able to elicit distinct shifts in steady-state fluoroquinolone accumulation, but we acknowledge that these non-specific inhibitors are unlikely to cover the extensive list of mycobacterial efflux pumps with their broad range of structural properties and substrate specificities.

First-line standard TB drugs ethambutol and rifampicin have much lower and higher molecular masses than ciprofloxacin respectively. Intracellular steady-state accumulation of both drugs by *M. bovis* BCG failed to decrease significantly upon polyamine pre-treatment, indicating that this polyamine-sensitive uptake process is more drug class specific than it is molecular mass-dependent. While linezolid and ciprofloxacin have comparable molecular masses, they have significantly differing hydrophobicity (ClogP of 0.168 and −0.725 respectively). Interestingly, polyamine treatment caused a 33% and 71% reduction in intracellular accumulation of hydrophobic linezolid and hydrophilic ciprofloxacin respectively. Intracellular uptake can be a combination of passive diffusion through the cell membrane and facilitated transport. Hence the opposite effects observed on fluoroquinolone and rifampicin uptake could have resulted from polyamine action through two distinct mechanisms. Although we were unable to confirm the type of interaction between polyamines and their target proteins in mycobacteria, we now understand that this interaction is reversible because saline washes enable the restoration of ciprofloxacin uptake. The positive charge of spermidine is an important determinant of its inhibitory mechanism. An increase in from 8 to 9 brought about a decrease in the inhibitory effects of spermidine by half. From the pK_a_ values of spermidine (8.34, 9.61 and 10.88) [24], one can calculate that the ratio of trivalent to divalent species is 10 fold higher at pH 8 than 9.

In *E. coli*, the inhibitory effect of polyamines on the outer membrane permeability is due to their specific interactions with the OmpF and OmpC porins [10]. Porins are trans-membrane proteins that act as channels for a wide variety of molecules. In facilitating chemotaxis and the flux of antibiotics, porins contribute significantly to the overall permeability of the outer membrane. There is general consensus that polyamine binding in *E. coli* causes a conformational change in the porin, which promotes the prolonged occupancy of the closed state, though it cannot be excluded that some steric blocking of the open channel may occur [18]. It is plausible that polyamines may inhibit mycobacterial porins in a similar manner. Although we have not demonstrated a direct interaction between polyamines and mycobacterial porins, several observations point towards a porin-mediated uptake mechanism for the fluoroquinolones. First, sensitivity to polyamine-treatment increases with increasing hydrophilicity, and second, inhibition of intracellular uptake by polyamines is dose- and pH-dependent, similar to the phenomenon observed with OmpF in *E. coli* [10], [18]. We therefore hypothesized that decreased outer membrane permeability in non-replicating *M. tuberculosis* is the result of decreased porin gene expression. The acquisition of drug tolerance due to decreased porin expression has been demonstrated in other bacterial species. Multiple antibiotic resistant (MAR) mutants of *E. coli*, for example, have reduced OmpF porins amongst other changes in their outer membrane. Several studies make reference to the starvation state and how changes in porin expression in *E. coli* aid in survival under stressful conditions [25], [26]. MspA is recognized as the major porin in *M. smegmatis*. It is responsible for nutrient and β-lactam influx [27], [28]. Hydrophilic fluoroquinolones have been shown to use Msp porins to enter *M. smegmatis* [6]. Interestingly, Hillman *et al* reported that MspA is barely detectable in *M. smegmatis* during the stationary phase of growth and that this species responds to glycerol starvation by shutting down *mspA* expression. It was suggested that other mycobacterial species respond similarly to nutrient starvation [29]. We compared the sensitivity of replicating and nutrient starved non-replicating *M. tuberculosis* to polyamines. We found that the treatment of actively replicating *M. tuberculosis* with spermidine reduced ciprofloxacin accumulation by half while non-replicating nutrient starved cultures showed marginal sensitivity to polyamines. We suggest that a reduction in this porin-mediated transport contributes to the phenotypic drug resistance to fluoroquinolones demonstrated by *M. tuberculosis* in the non-replicating state.

To our knowledge, no attempt has yet been made to characterize changes in porin expression levels with respect to the growth state of *M. tuberculosis*. This challenge is compounded by the limited identification and characterization of OMPs of this species. OmpATb (Rv0899) was the first suggested porin in *M. tuberculosis* [30]. This has since been disputed by structural and functional studies [31], [32]. Bioinformatics approaches have been undertaken to predict OMPs of *M. tuberculosis*. The combination of secondary structure prediction and the computation of amphiphilicity allowed for the genome-wide identification of putative OMPs. Two hypothetical proteins, Rv1698 and Rv1973 were identified [22]. Mah *et al's* search for outer membrane pore proteins led to further refinement of this algorithm, enabling the additional identification of a range on putative OMPs from seven mycobacterial species. These include Rv1968, Rv1970, Rv1351, Rv1352, Rv2270, Rv0431 and Rv0227 [21]. We pursued the study of gene expression of these ten OMPs in replicating and non–replicating bacteria in order to identify the cause of decreased drug permeability of nutrient-starved cells.

*rv1698* was identified as being severely under-expressed (≥25 fold) during nutrient starvation. Rv1698 was recently profiled as a channel-forming protein in *M. tuberculosis*. In a previous study, a *M. smegmatis* strain with the major porin gene *mspA* knocked out displayed significant decreases in susceptibility to ampicillin, chloramphenicol and cephaloridine. Susceptibility of this ΔmspA mutant to these β-lactams was partially restored by the expression of *rv1698* [33]. We confirmed that a half-hour incubation period with spermidine does not have a significant effect on the expression levels of *rv1698* (results not shown), supporting our hypothesis that spermidine interacts directly with the mycobacterial porin protein to reduce net drug permeability. Rv0431 also displayed significant under-expression in non-replicating culture. Rv1698-like proteins are only found in the suborder Corynebacterineae which includes mycolic acid–containing bacteria such as mycobacteria [33]. Interestingly, Rv0431 orthologues contain a domain that is also unique to genera of Corynebacterineae [22]. Several OMPs, including *ompATb,* displayed significant increase in expression in non-replicating cultures. The upregulation of Rv1352 and Rv2270 expression was also reported by Betts *et al* in their genome-wide protein expression analysis of nutrient-starved *M. tuberculosis* [12]. Our analyses suggest that Rv1698 may contribute to fluoroquinolone transport in *M. tuberculosis* and that a reduction in its expression in non–replicating bacteria brings about decreased fluoroquinolone susceptibility. The additional possibility that transport proteins of *M. tuberculosis* may be regulated by endogenous molecules in response to unfavorable conditions, as observed with *E.coli* [34], awaits further investigation.

It has been hypothesized that the development of fluoroquinolone resistance in some species may be the result of diminished drug uptake processes acting in conjunction with enhanced efflux capabilities [35]. We have demonstrated that spermidine concentrations of 1 to 2.5 mM rescue *M. bovis* BCG from ciprofloxacin bactericidal activity. These concentrations are below the spermidine MIC of 4 mM. It should be noted however that partial growth inhibition was observed at 2.5 mM while growth remained unaffected at 1 mM spermidine. Thus it is possible that partial growth inhibition may have contributed to the reduced killing activity of ciprofloxacin in the presence of 2.5 mM spermidine. Nevertheless, the dose-proportional response we observe between 1 and 2.5 mM spermidine (Figure 4) supports the notion that transport processes play an important role in maintaining fluoroquinolone susceptibility in mycobacteria. The various drug resistance mechanisms in other bacteria include decreased synthesis, mutation, inhibition by endogenous molecules, and internalization and degradation of proteins that facilitate drug uptake [36]. We suggest that such mechanisms may also be responsible for fluoroquinolone resistance acquisition in mycobacteria. While there is evidence of cell wall thickening in *M. tuberculosis* upon the onset of dormancy, it remains unclear how this affects the orientation and function of outer membrane proteins [37].

The inhibition of drug uptake by polyamines has *in vivo* implications as well. Proliferating and infected tissue reportedly have increased levels of polyamines such as spermine [38]–[40]. Interestingly, Hirsh and Dubos first reported increased local spermine levels at sites of mycobacterial infection over 60 years ago [41]. They suggested that spermine possesses tuberculostatic activity *in vivo* that is distinct from conventional immune processes. It was later shown that human monocytes express a non-selective polyamine transporter, and that increased monocyte uptake of spermidine upon stimulation plays a role in the modulation of inflammatory responses. In light of our recent observation that polyamines can reduce the overall drug permeation of *M. tuberculosis*, it appears that endogenous polyamines in eukaryotic macrophages may contribute towards the development of dormancy and phenotypic drug resistance of the intracellular tubercle bacillus.

## References

[1] Dye C, Williams BG (2010) The population dynamics and control of tuberculosis. Science 328: 856–861. 20466923 10.1126/science.1185449

[2] De Rossi E, Ainsa JA, Riccardi G (2006) Role of mycobacterial efflux transporters in drug resistance: an unresolved question. Fems Microbiol Rev 30: 36–52. 16438679 10.1111/j.1574-6976.2005.00002.x

[3] Liu J, Takiff HE, Nikaido H (1996) Active efflux of fluoroquinolones in *Mycobacterium smegmatis* mediated by LfrA, a multidrug efflux pump. J Bacteriol 178: 3791–3795. 8682782 10.1128/jb.178.13.3791-3795.1996 PMC232638

[4] Pasca MR, Guglierame P, Arcesi F, Bellinzoni M, De Rossi E, et al (2004) Rv2686c-Rv2687c-Rv2688c, an ABC fluoroquinolone efflux pump in *Mycobacterium tuberculosis* . Antimicrob Agents Chemother 48: 3175–3178. 15273144 10.1128/AAC.48.8.3175-3178.2004 PMC478549

[5] Haemers A, Leysen DC, Bollaert W, Zhang MQ, Pattyn SR (1990) Influence of N Substitution on Antimycobacterial Activity of Ciprofloxacin. Antimicrob Agents Chemother 34: 496–497. 2334166 10.1128/aac.34.3.496 PMC171627

[6] Danilchanka O, Pavlenok M, Niederweis M (2008) Role of porins for uptake of antibiotics by *Mycobacterium smegmatis* . Antimicrob Agents Chemother 52: 3127–3134. 18559650 10.1128/AAC.00239-08 PMC2533485

[7] Tabor CW, Tabor H (1985) Polyamines in microorganisms. Microbiol Rev 49: 81–99. 3157043 10.1128/mr.49.1.81-99.1985 PMC373019

[8] Iyer R, Delcour AH (1997) Complex inhibition of OmpF and OmpC bacterial porins by polyamines. J Biol Chem 272: 18595–18601. 9228026 10.1074/jbc.272.30.18595

[9] Chevalier J, Mallea M, Pages JM (2000) Comparative aspects of the diffusion of norfloxacin, cefepime and spermine through the F porin channel of *Enterobacter cloacae* . Biochem J 348: 223–227. 10794735 PMC1221057

[10] Dela Vega AL, Delcour AH (1996) Polyamines decrease *Escherichia coli* outer membrane permeability. J Bacteriol 178: 3715–3721. 8682771 10.1128/jb.178.13.3715-3721.1996 PMC232627

[11] Samartzidou H, Delcour AH (1999) Excretion of endogenous cadaverine leads to a decrease in porin-mediated outer membrane permeability. J Bacteriol 181: 791–798. 9922241 10.1128/jb.181.3.791-798.1999 PMC93444

[12] Betts JC, Lukey PT, LRobb LC, McAdam RA, Duncan K (2002) Evaluation of a nutrient starvation model of *Mycobacterium tuberculosis* persistence by gene and protein expression profiling. Mol Microbiol 43: 717–731. 11929527 10.1046/j.1365-2958.2002.02779.x

[13] Gengenbacher M, Rao SP, Pethe K, Dick T (2010) Nutrient-starved, non-replicating *Mycobacterium tuberculosis* requires respiration, ATP synthase and isocitrate lyase for maintenance of ATP homeostasis and viability. Microbiology 156: 81–87. 19797356 10.1099/mic.0.033084-0

[14] Xie Z, Siddiqi N, Rubin EJ (2005) Differential antibiotic susceptibilities of starved *Mycobacterium tuberculosis* isolates. Antimicrob Agents Chemother 49: 4778–4780. 16251329 10.1128/AAC.49.11.4778-4780.2005 PMC1280169

[15] Seiler P, Ulrichs T, Bandermann S, Pradl L, Jorg S, et al (2003) Cell-wall alterations as an attribute of *Mycobacterium tuberculosis* in latent infection. J Infect Dis 188: 1326–1331. 14593589 10.1086/378563

[16] Piddock LJ, Ricci V (2001) Accumulation of five fluoroquinolones by *Mycobacterium tuberculosis* H37Rv. J Antimicrob Chemother 48: 787–791. 11733462 10.1093/jac/48.6.787

[17] Livak KJ, Schmittgen TD (2001) Analysis of relative gene expression data using real-time quantitative PCR and the 2(T)(-Delta Delta C) method. Methods 25: 402–408. 11846609 10.1006/meth.2001.1262

[18] Dela Vega AL, Delcour AH (1995) Cadaverine induces closing of *E. coli* porins. The EMBO journal 14: 6058–6065. 8846798 10.1002/j.1460-2075.1995.tb00294.x PMC394726

[19] Pasca MR, Guglierame P, De Rossi E, Zara F, Riccardi G (2005) mmpL7 gene of *Mycobacterium tuberculosis* is responsible for isoniazid efflux in *Mycobacterium smegmatis* . Antimicrob Agents Chemother 49: 4775–4777. 16251328 10.1128/AAC.49.11.4775-4777.2005 PMC1280163

[20] Silva PE, Bigi F, Santangelo MP, Romano MI, Martin C, et al (2001) Characterization of P55, a multidrug efflux pump in *Mycobacterium bovis* and *Mycobacterium tuberculosis.* . Antimicrob Agents Chemother 45: 800–804. 11181364 10.1128/AAC.45.3.800-804.2001 PMC90377

[21] Mah N, Perez-Iratxeta C, Andrade-Navarro MA (2010) Outer membrane pore protein prediction in mycobacteria using genomic comparison. Microbiology 156: 2506–2515. 20466765 10.1099/mic.0.040089-0

[22] Song H, Sandie R, Wang Y, Andrade-Navarro MA, Niederweis M (2008) Identification of outer membrane proteins of *Mycobacterium tuberculosis* . Tuberculosis (Edinb) 88: 526–544. 18439872 10.1016/j.tube.2008.02.004 PMC2615007

[23] Basle A, Delcour AH (2004) Regulation of Bacterial Porin Function. In: Benz R, editor. Bacterial and Eukaryotic Porins: Structure, Function and Mechanism. Germany: Wiley-VCH. pp. 79–98.

[24] Rock DM, MacDonald RL (1992) Spermine and related polyamines produce a voltage-dependent reduction of N-methyl-D-aspartate receptor single-channel conductance. Mol Pharmacol 42: 157–164. 1378923

[25] Darcan C, Ozkanca R, Idil O, Flint KP (2009) Viable but non-culturable state (VBNC) of *Escherichia coli* related to EnvZ under the effect of pH, starvation and osmotic stress in sea water. Pol J Microbiol 58: 307–317. 20380141

[26] Ozkanca R, Flint KP (2002) The effect of starvation stress on the porin protein expression of *Escherichia coli* in lake water. Lett Appl Microbiol 35: 533–537. 12460439 10.1046/j.1472-765x.2002.01230.x

[27] Mailaender C, Reiling N, Engelhardt H, Bossmann S, Ehlers S, et al (2004) The MspA porin promotes growth and increases antibiotic susceptibility of both *Mycobacterium bovis* BCG and *Mycobacterium tuberculosis* . Microbiology 150: 853–864. 15073295 10.1099/mic.0.26902-0

[28] Stephan J, Bender J, Wolschendorf F, Hoffmann C, Roth E, et al (2005) The growth rate of *Mycobacterium smegmatis* depends on sufficient porin-mediated influx of nutrients. Mol Microbiol 58: 714–730. 16238622 10.1111/j.1365-2958.2005.04878.x

[29] Hillmann D, Eschenbacher I, Thiel A, Niederweis M (2007) Expression of the major porin gene mspA is regulated in *Mycobacterium smegmatis* . J Bacteriol 189: 958–967. 17142388 10.1128/JB.01474-06 PMC1797333

[30] Senaratne RH, Mobasheri H, Papavinasasundaram KG, Jenner P, Lea EJ, et al (1998) Expression of a gene for a porin-like protein of the OmpA family from *Mycobacterium tuberculosis* H37Rv. J Bacteriol 180: 3541–3547. 9657995 10.1128/jb.180.14.3541-3547.1998 PMC107320

[31] Song H, Huff J, Janik K, Walter K, Keller C, et al (2011) Expression of the ompATb operon accelerates ammonia secretion and adaptation of *Mycobacterium tuberculosis* to acidic environments. Mol Microbiol 80: 900–918. 21410778 10.1111/j.1365-2958.2011.07619.x PMC3091969

[32] Teriete P, Yao Y, Kolodzik A, Yu J, Song H, et al (2010) *Mycobacterium tuberculosis* Rv0899 adopts a mixed alpha/beta-structure and does not form a transmembrane beta-barrel. Biochemistry 49: 2768–2777. 20199110 10.1021/bi100158s PMC2847638

[33] Siroy A, Mailaender C, Harder D, Koerber S, Wolschendorf F, et al (2008) Rv1698 of *Mycobacterium tuberculosis* represents a new class of channel-forming outer membrane proteins. J Biol Chem 283: 17827–17837. 18434314 10.1074/jbc.M800866200 PMC2440620

[34] Samartzidou H, Mehrazin M, Xu ZH, Benedik MJ, Delcour AH (2003) Cadaverine inhibition of porin plays a role in cell survival at acidic pH. J Bacteriol 185: 13–19. 12486035 10.1128/JB.185.1.13-19.2003 PMC141942

[35] Hooper DC (2003) Mechanisms of quinolone resistance. In: Hooper DC, Rubinstein E, editors. Quinolone Antimicrobial Agents. Washington: ASM Press. pp. 41–68.

[36] Pages JM, James CE, Winterhalter M (2008) The porin and the permeating antibiotic: a selective diffusion barrier in Gram-negative bacteria. Nat Rev Microbiol 6: 893–903. 18997824 10.1038/nrmicro1994

[37] Cunningham AF, Spreadbury CL (1998) Mycobacterial stationary phase induced by low oxygen tension: cell wall thickening and localization of the 16-kilodalton alpha-crystallin homolog. J Bacteriol 180: 801–808. 9473032 10.1128/jb.180.4.801-808.1998 PMC106957

[38] Babbar N, Gerner EW (2011) Targetting polyamines and inflammation for cancer prevention. In: Senn HJ, Otto F, editors. Clinical Cancer Prevention. Berlin: Springer. pp. 49–64 10.1007/978-3-642-10858-7_4 PMC3587145 21253788

[39] Clarke JR, Tyms AS (1991) Polyamine biosynthesis in cells infected with different clinical isolates of human cytomegalovirus. J Med Virol 34: 212–216. 1658212 10.1002/jmv.1890340403

[40] Colombatto S, De Agostini M, Corsi D, Sinicco A (1989) Polyamines in lymphocytes from patients infected by human immunodeficiency virus. Biol Chem Hoppe Seyler 370: 745–748. 2505811 10.1515/bchm3.1989.370.2.745

[41] Hirsch JG, Dubos RJ (1952) The effect of spermine on tubercle bacilli. J Exp Med 95: 191–208. 14907970 10.1084/jem.95.2.191 PMC2212060

